# Supplementary material for: Comparative Genomics of Peroxisome Biogenesis Proteins: Making Sense of the PEX Proteins
Source: Front Cell Dev Biol. 2021 May 20;9:654163. doi: 10.3389/fcell.2021.654163 (PMC8172628; doi:10.3389/fcell.2021.654163)
Supplement: Supplementary file 1 [file Data_Sheet_1.pdf]

Supplementary Material

1 Supplementary Data

1.1 Supplementary Figures

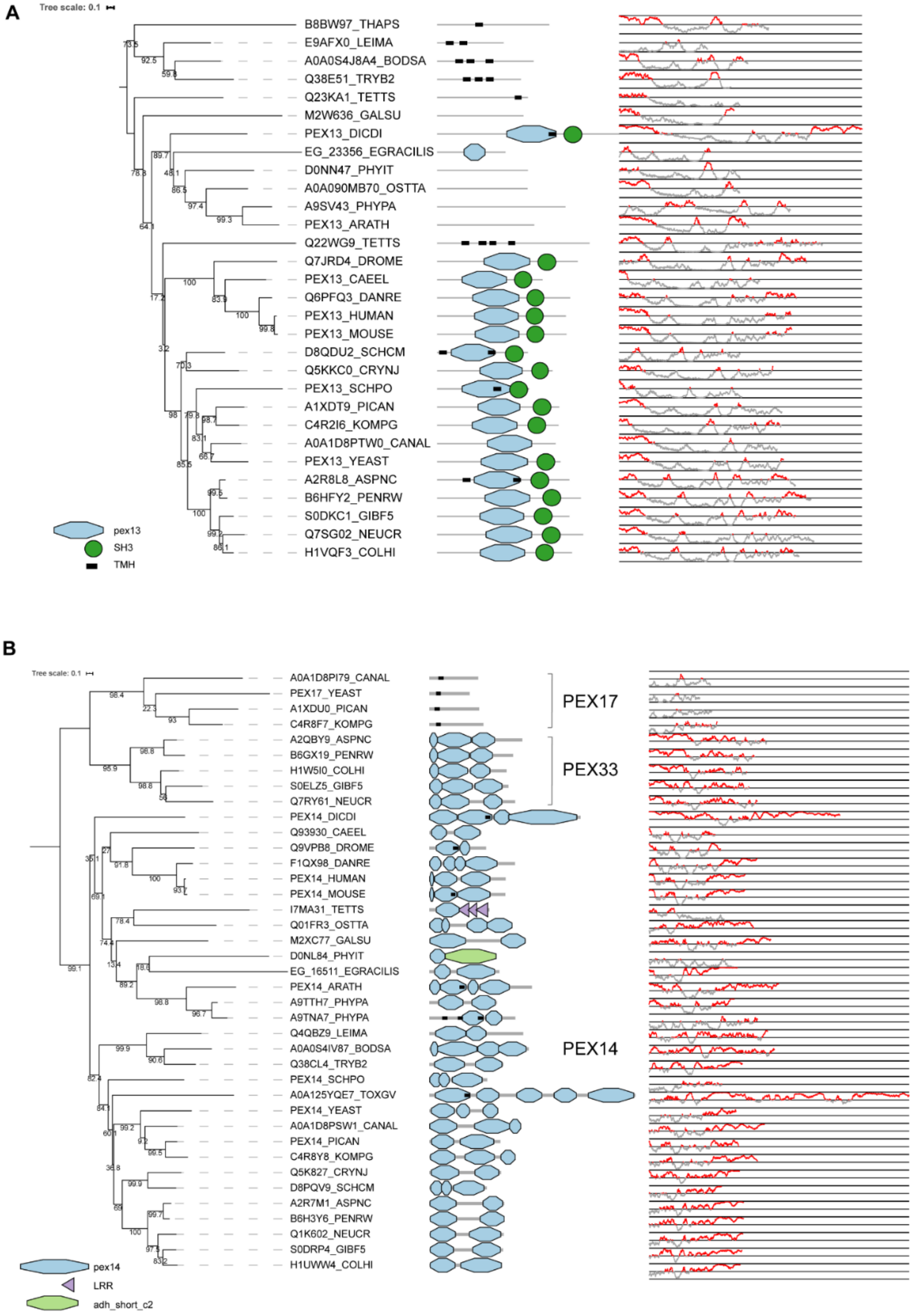

Figure S1: Phylogeny and protein features of A) PEX13 and B) PEX14/17/33 orthologs. The phylogeny is rooted at mid-point to ease the visualization. Note that the topology does not necessarily reflect the actual evolutionary trajectory of such proteins. Protein domain architecture is defined by pfam annotations and transmembrane helix according to TMHMM software. The line-dot plot, indicates the regions predicted to be disordered (red) and not disordered (grey).

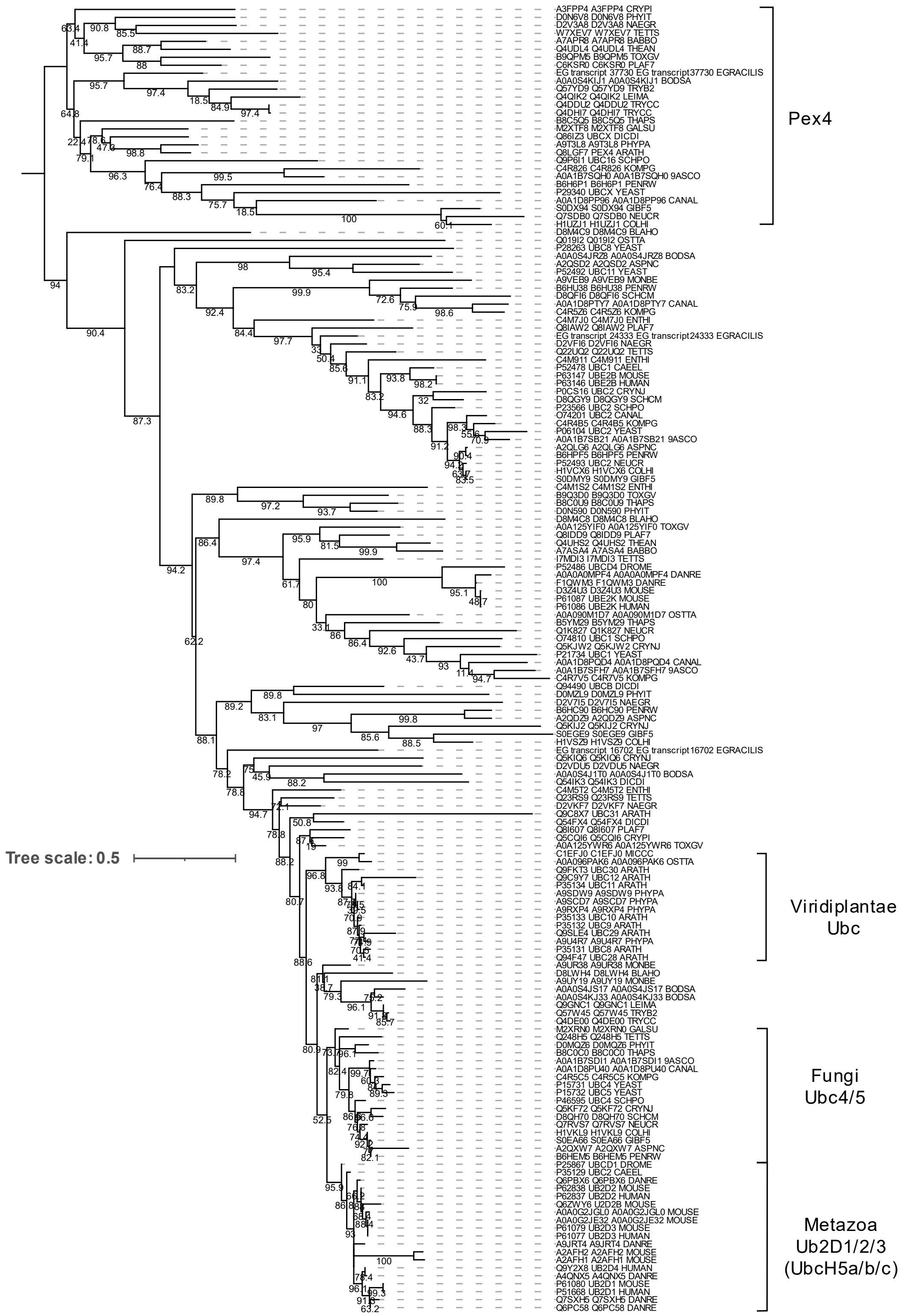

Figure S2: Phylogeny of the ubiquitin-conjugating enzymes closest to PEX4. The phylogeny is rooted at mid-point to ease the visualization leaving the Pex4 subfamily separated from the rest. The closest homologs to PEX4 were retrieved with an e-value threshold of 1e-15 including several members of the ubiquitin-conjugating enzymes such as the PEX4 functional homolog in Metazoa UbcH5a/b/c (UbcD1/2/3 in the figure) and their co-orthologs Ubc4/5 in Fungi.

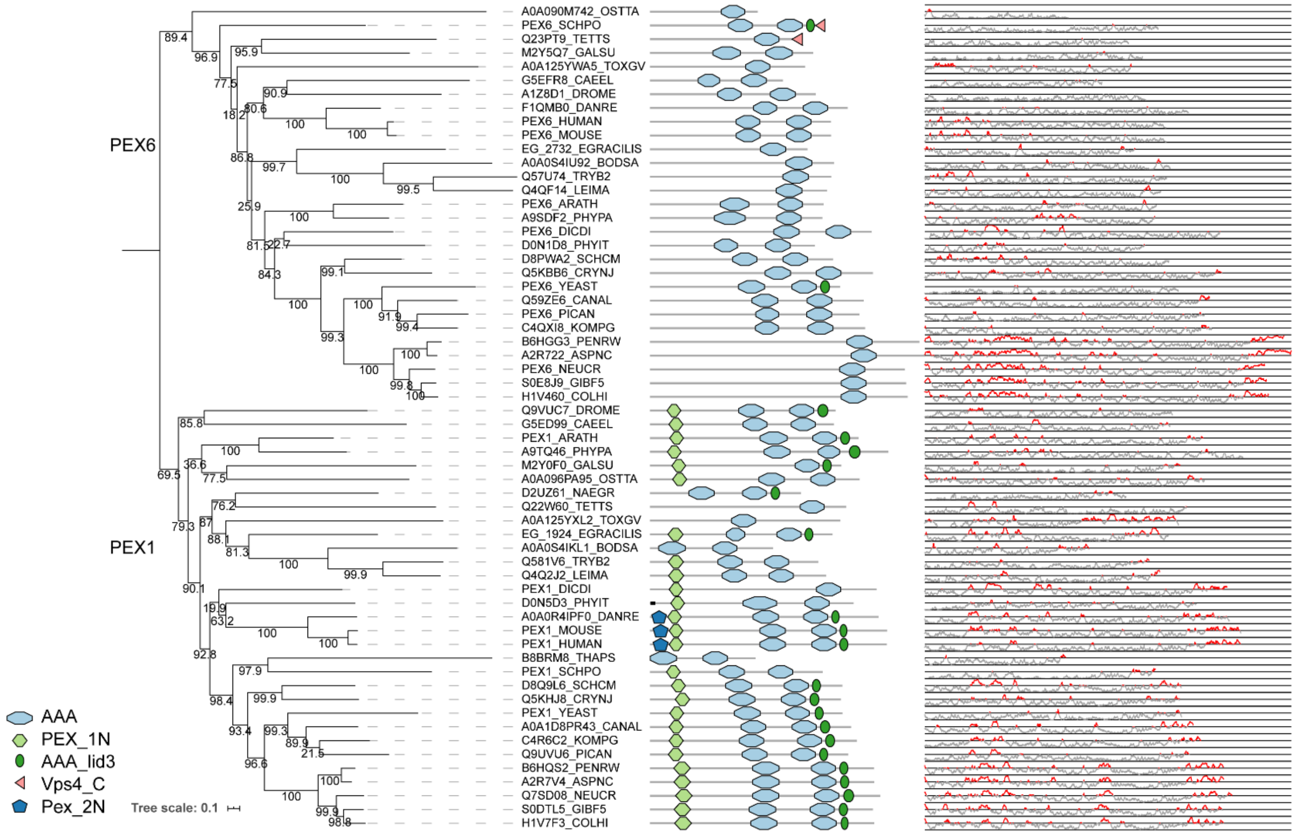

Figure S3: Phylogeny and protein features of PEX1/6 orthologs. The phylogeny is rooted at mid-point to ease the visualization. Note that the topology does not necessarily reflect the actual evolutionary trajectory of such proteins. Protein domain architecture is defined by pfam annotations and transmembrane helix according to TMHMM software. The line-dot plot, indicates the regions predicted to be disordered (red) and not disordered (grey).

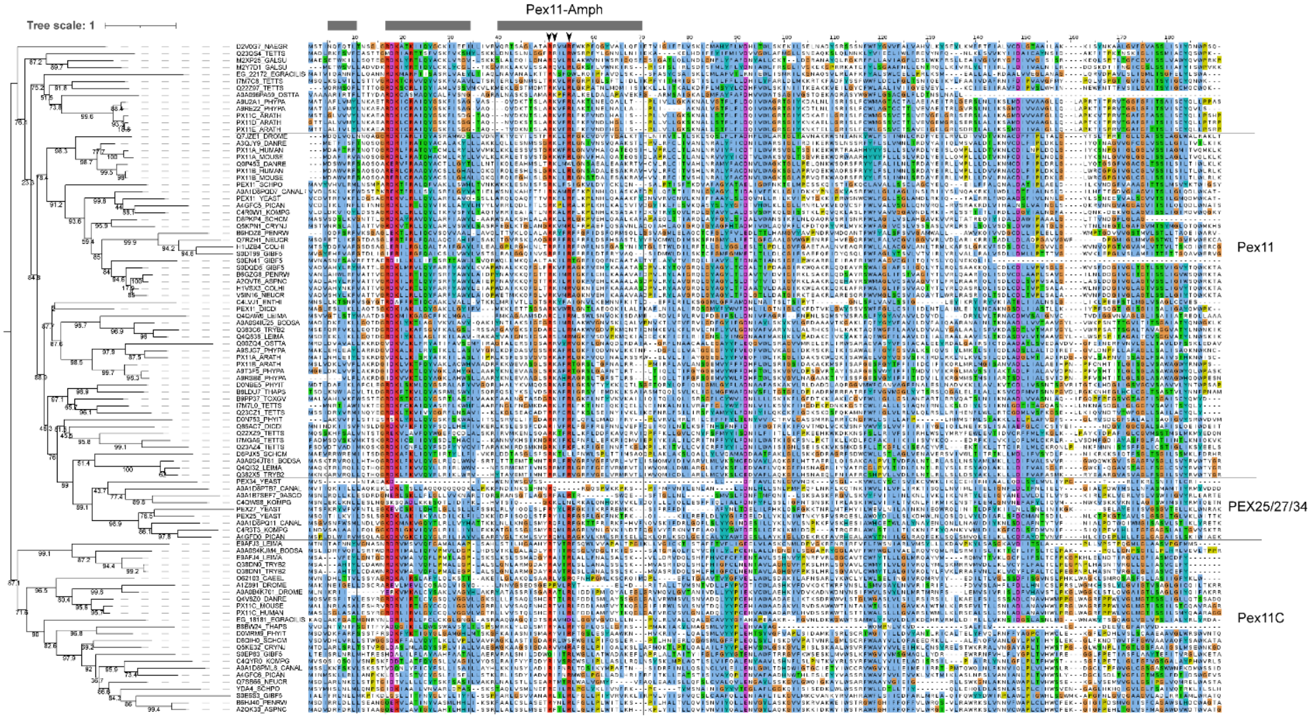

Figure S4: Multiple sequence alignment of Pex11 family proteins. Grey bars above sequences denote predicted α-helices and the N-terminal amphipathic helix (Pex11-Amph). Residues are coloured based on physico-chemical properties according to ClustalW. The phylogeny is rooted at mid-point to ease the visualization. Note that the topology does not necessarily reflect the actual evolutionary trajectory of such proteins.

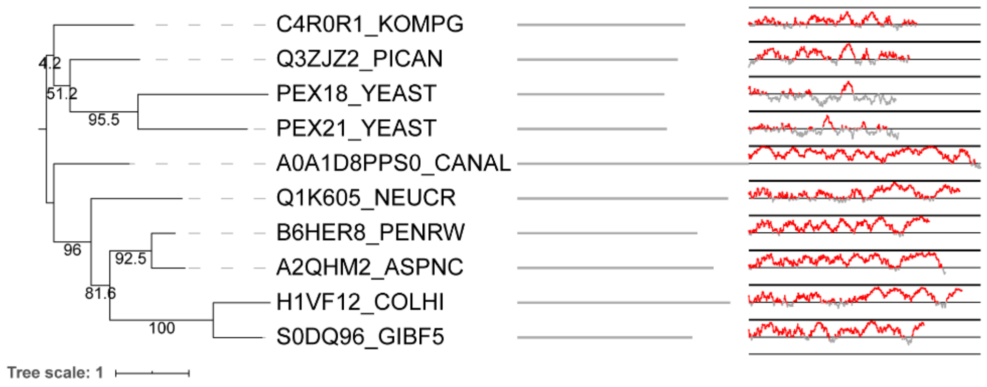

Figure S5: Phylogeny and protein features of PEX118/20/21 orthologs. The phylogeny is rooted at mid-point to ease the visualization and labels of the main taxonomic groups are coloured accordingly to the legend. Note that the topology does not necessarily reflect the actual evolutionary trajectory of such proteins. Protein domain architecture is defined by pfam annotations and transmembrane helix according to TMHMM software. The line-dot plot, indicates the regions predicted to be disordered (red) and not disordered (grey).
